# 3DICE: interpretable 3D cross-modal learning for drug–target interaction prediction and large-scale drug discovery

**DOI:** 10.1093/bioinformatics/btag488

**Published:** 2026-07-02

**Authors:** Austin Zi Rui Liu, Nguyen Quoc Khanh Le, Matthew Chin Heng Chua

**Affiliations:** Raffles Institution, Singapore, 575954, Singapore; AIBioMed Lab, Taipei Medical University, Taipei, 110, Taiwan; AIBioMed Lab, Taipei Medical University, Taipei, 110, Taiwan; In-Service Master Program in Artificial Intelligence in Medicine, College of Medicine, Taipei Medical University, Taipei, 110, Taiwan; Translational Imaging Research Center, Taipei Medical University Hospital, Taipei, 110, Taiwan; Department of Biomedical Informatics, Yong Loo Lin School of Medicine, National University of Singapore, Singapore, 119615, Singapore

## Abstract

**Motivation:**

Drug–target interaction (DTI) prediction is a crucial step in modern drug discovery. Accurate and efficient predictions can substantially reduce costs and development time. Applications of deep learning methods for this purpose have been extensively studied in recent years, yielding instrumental contributions to this field. However, existing methods face issues pertaining to efficient learning of drug and target feature representations, which is detrimental to generalizability and performance in cold-start scenarios. Most approaches extract representations from SMILES strings for drugs and FASTA sequences for target proteins, which encode limited 3D structural information. Additionally, many models lack explainability, being black boxes that provide little physical insight into the underlying mechanisms behind such interactions.

**Results:**

We propose 3DICE, a novel framework leveraging co-attention-based fusion and massively pre-trained 3D structural encoders for both drugs and proteins. Uni-Mol and ESM-IF1 are employed to generate high-fidelity, 3D structure-aware embeddings which enable richer geometric and chemical understanding. Cross-modal fusion modules further augment representations to model intermolecular binding relationships. Importantly, this mechanism also provides intrinsic interpretability, highlighting and enabling qualitative analysis of most influential atoms or residues. Experiments conducted on two canonical benchmark datasets display the competitiveness of our model in real-world scenarios. 3DICE outperformed state-of-the-art models across multiple metrics on the DrugBank and KIBA datasets. Additional experiments provide a more rigorous analysis of interpretability than is typically reported in prior DTI studies, and we find that attention consistently highlights decision-critical regions which is not intrinsically class-specific.

**Availability:**

Our model and dataset are freely available at: https://github.com/austinatose/3DICE.

## 1 Introduction

Drug–target interaction (DTI) prediction is a crucial aspect of modern drug discovery Yamanishi *et al.* (2008). Experimental screening of drug–target interactions is expensive, slow, and failure-prone (Paul *et al.* 2010, DiMasi *et al.* 2016). In-silico methods help to whittle down the vast search space to a manageable number of DTIs for experimental testing and verification (Vefghi *et al.* 2025, Ru *et al.* 2025). Despite decades of research, accurate computational DTI prediction remains challenging. The many-to-many nature of DTIs, exacerbated by the chemical diversity of small molecules and structural diversity of proteins, complicates methods (Mayr *et al.* 2018). Sparse, noisy and biased experimental data also need to be carefully handled (Xia 2017). Several studies have explored docking and physics-based methods, which are accurate and explainable, but are traditionally computationally intensive (Sliwoski *et al.* 2014, Mobley and Gilson 2017), although recent advances have enabled high-throughput virtual screening at scale Fraser *et al.* (2025), suggesting that scalability alone is not a decisive limitation. Nevertheless, physics-based methods remain constrained by the accuracy of scoring functions, sensitivity to structural quality, and the difficulty of modelling induced fit and entropic effects.

In recent years, machine learning techniques have emerged as an efficient data-driven alternative (Chen *et al.* 2018, Öztürk *et al.* 2018). The approach taken in DTI prediction works fall within the broader paradigm of proteochemometric (PCM) modelling (Qiu *et al.* 2017, Bongers *et al.* 2019), which jointly represents both ligand and target features to model bioactivity across multiple compound–protein pairs. Originally formulated using classical descriptors and statistical models, PCM has since evolved substantially; recent deep learning-based DTI methods, including 3DICE, can be viewed as a modern instantiation of this paradigm, replacing hand-crafted descriptors with learned representations.

As such, a key component of computational DTI prediction is learning representations for both drugs and target proteins. In classical PCM approaches, molecular fingerprints derived from the molecular graph (such as ECFP, Rogers and Hahn 2010) served as the dominant drug representation, while protein descriptors were typically computed from sequence or physicochemical properties (Li *et al.* 2006). With the advent of deep learning, approaches also started to rely on character-level embeddings of Simplified Molecular Input Line System (SMILES) strings for drug molecules, and FASTA, or amino acid sequences for proteins. Early models such as DeepDTA (Öztürk *et al.* 2018) and DeepCPI (Wan *et al.* 2019) adopt this approach, and similar representations continue to be used by more recent methods like MolTrans (Huang *et al.* 2021). While effective in capturing some chemical and sequential patterns, both fingerprint- and sequence-based representations neglect the 3D geometry that fundamentally governs binding.

There have been attempts to better capture molecular structure. Graph-based neural networks (GNNs) model geometry through atom-bond graphs. GraphDTA (Nguyen *et al.* 2021) and GraphCPI (Quan *et al.* 2019) use GNNs encoding drug molecular graphs, while TransformerCPI (Chen *et al.* 2020) uses Graph Convolutional Networks (GCNs). Graphormer (Gao *et al.* 2024) further embeds molecular graphs into vector representations for use in its transformer architecture. In contrast, explicit modelling of protein structures remains comparatively limited, with works citing complexity and demanding computational requirements (Zhao *et al.* 2025). Only a small number of works, such as GraphDTI (Liu *et al.* 2021), attempt to encode protein structures.

In parallel, large-scale pretrained models have also emerged as a promising alternative in extracting protein and drug features due to their strong generalization capabilities (Lee *et al.* 2024, Sun *et al.* 2024, Zhang *et al.* 2024, Zhao *et al.* 2024, 2025). On the drug side, sequence-based ChemBERTa and graph-based MG-BERT (Zhang *et al.* 2021) have been established. On the protein side, the ProtTrans (Elnaggar *et al.* 2022) and Evolutionary Scale Modelling (ESM) (Lin *et al.* 2023) transformers have demonstrated strong performance on downstream tasks. The EviDTI (Zhao *et al.* 2024) framework adopts this paradigm, with proteins processed with ProtTrans and drugs with MG-BERT, augmented with geometric representations from GeoGNN. However, current methods still remain largely sequence or topology-driven and do not explicitly encode full 3D structural information that is pertinent in binding.

A model that stands out is Uni-Mol (Zhou *et al.* 2023), which introduces a large-scale 3D Molecular Representation Learning framework that allows it to learn structure-aware embeddings from conformers. Uni-Mol has started to see adoption in related works including MocFormer (Zhang *et al.* 2024) and DrugCLIP (Jia *et al.* 2026), showcasing its potential and versatility. Complementary to this, Evolutionary Scale Modelling—Inverse Folding 1 (ESM-IF1) (Hsu *et al.* 2022) is a unique pretrained protein structural encoder. Originally designed for predicting protein sequences based on 3D backbone coordinates, it has the novel ability to produce embeddings encoding rich geometric and spatial relationships between protein residues given the 3D protein structure. To the best of our knowledge, ESM-IF1 has not been previously explored in the context of DTI prediction. Despite its strong theoretical potential, its adoption may have been limited due to the scarcity of experimentally resolved protein structures and the substantial preprocessing required. However, recent advances in large-scale protein structure prediction, most notably AlphaFold (Jumper *et al.* 2021), substantially mitigate this limitation by enabling training on predicted protein structures. These advances together demonstrate that new possibilities for high-fidelity representations for both drugs and proteins are now feasible through the coupling of 3D structure-awareness with the strong generalization capabilities afforded by massive pretraining.

In a different vein, cross-modal fusion is an integral component of DTI modelling, as final prediction ultimately requires a joint representation of drug and protein features. While a simple concatenation of independently encoded and augmented representations can be used to form such a joint vector (Öztürk *et al.* 2018, Chen *et al.* 2020, Liu *et al.* 2021, Nguyen *et al.* 2021, Zhao *et al.* 2025), from a physical perspective, the interactions are inherently governed by specific local interactions between drugs and proteins, which makes explicit modelling of this qualitatively crucial. Many models have sought to do so. MolTrans utilizes a Hadamard-product-based pooling; DrugBAN (Bai *et al.* 2023) a bilinear attention network; and MocFormer bilinear pooling. More recently, cross-attention-based (Chen *et al.* 2021) approaches have been proposed, including ICAN (Kurata and Tsukiyama 2022), CAT-DTI (Zeng *et al.* 2024), and CAIHGNN (Che *et al.* 2026). In addition to improving predictive performance, such explicit interaction modelling also facilitates interpretability, as learned attention weights or pairwise interaction maps can be analysed to identify the key binding sites for better physical understanding.

In this work, we propose 3DICE, a novel and interpretable framework for DTI prediction. 3DICE leverages massively pretrained 3D structural encoders, namely ESM-IF1 for proteins and Uni-Mol for drugs, to produce high-fidelity, information-rich representations. Additionally, the framework is explicitly designed to model drug-protein interactions through a co-attention mechanism (Lu *et al.* 2016), which enables both improved predictive performance and intrinsic interpretability by highlighting regions critical to interactions.

While conceptually related works do exist, 3DICE is distinguished from them in several important respects. MocFormer (Zhang *et al.* 2024) similarly pairs Uni-Mol drug embeddings with a pretrained protein encoder, but relies on ESM-2, a sequence-based model, rather than a 3D structural encoder on the protein side, and employs bilinear pooling rather than explicit co-attention for interaction modelling. DrugCLIP (Jia *et al.* 2026) also leverages Uni-Mol on the ligand side within a contrastive learning framework, alongside 3D protein pocket embeddings extracted from local binding site structures. However, it is designed for the related but distinct task of pocket-based virtual screening, where a pre-defined binding pocket must be supplied as input, in contrast to the end-to-end DTI setting addressed here in which no prior knowledge of binding site location is assumed. To the best of our knowledge, 3DICE is therefore the first end-to-end DTI framework to jointly employ pretrained 3D structural encoders on both the drug and protein sides, additionally within an explicitly interpretable interaction modelling architecture.

Extensive experiments demonstrate that 3DICE achieves competitive performance across multiple benchmark datasets. We further perform comprehensive analyses of robustness, interpretability, and learned interaction patterns, showing that 3DICE provides an effective and interpretable framework for DTI prediction.

## 2 Materials and methods

### 2.1 Architecture

**Figure 1:**
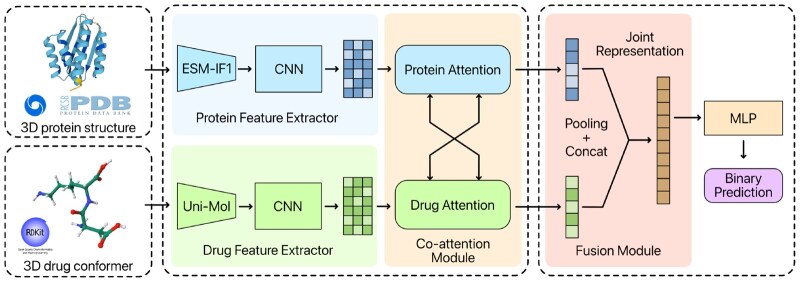
3DICE Framework.

Figure 1 shows the proposed 3DICE framework. The framework comprises five main components: a protein feature extractor, a drug feature extractor, a co-attention module, a fusion module, followed by a readout multi-layer perceptron (MLP). In the protein feature extractor, the pre-trained model ESM-IF1 was used to encode 3D protein features given the 3D spatial structures. A 2-layer 1D CNN across the sequence dimension was used to extract local substructural patterns. In the drug feature extractor, the pre-trained model Uni-Mol was used to capture 3D stereochemical information from drug conformers. A similar CNN to that on the protein side was implemented to augment drug features. Next, the enhanced protein and drug representations are fed into a co-attention module. By attending protein residues to drug atoms and vice versa, the module explicitly considers bidirectional cross-modal binding interactions. The embeddings are then pooled, reduced in dimension with a 1-layer MLP and concatenated to form a joint representation of each DTI pair. This is forwarded into a fully connected MLP to produce the final prediction. Detailed information about the framework is outlined in Appendix B, available as supplementary data at *Bioinformatics* online.

### 2.2 Datasets

#### 2.2.1 Details

The DrugBank (Wishart *et al.* 2018) and KIBA (Tang *et al.* 2014) datasets provide drug and protein identification codes and canonical string representations (SMILES and FASTA), which are customarily used in literature. However, in this study, 3D structural information is required to generate the embeddings used. Protein UniProt IDs were cross-referenced with the Protein Data Bank (PDB) (Berman *et al.* 2000). Experimentally obtained structures with the highest fidelity selected based on resolution were identified and fetched in mmCIF format. When experimental structures were unavailable, structures predicted by AlphaFold (Jumper *et al.* 2021) were used. These were obtained from AlphaFoldDB (Fleming *et al.* 2025). Drug 3D conformers were generated from SMILES strings using RDKit’s ETKDG algorithm (Riniker and Landrum 2015) followed by MMFF94 force-field optimization (Halgren 1996). The resultant molecular coordinates were used for downstream tasks.

When SMILES strings could not be parsed or if 3D protein information was unavailable from both PDB and AlphaFoldDB, the affected drug–protein pairs were removed. 3DICE itself does not face character length, size or structural complexity limits. However, the baseline models used for benchmarking may have such constraints. To ensure a fair comparison, the datasets were further pruned down to the smallest common subset that is supported by all the models. This involved removing drugs with inorganic compounds or very small molecule compounds (compounds with heavy atom count <6 or molecular weight <100 g/mol), and the maximum length of protein FASTA strings was limited to 700.

To split our dataset, we first cluster all unique drugs by their ECFP4 Morgan fingerprint Tanimoto similarity using the Butina algorithm Butina (1999), and cluster all unique proteins using PyMMseqs. Each interaction pair is then assigned a combined identity. Finally, these pair-groups are distributed across train, validation, and test sets using a greedy bin-packing algorithm to an 8:1:1 ratio, such that no combined drug-cluster/protein-cluster group appeared in more than one split.

We additionally constructed cold-start scenarios to assess the model’s ability to predict novel DTIs in realistic drug discovery settings, where the same splitting strategy as above was used but with the additional constraint that the same protein or drug cannot appear in multiple splits.

#### 2.2.2 DrugBank

DrugBank 5.1.13 (Wishart *et al.* 2018), published on January 2, 2025, was used in this study. In total, 13 098 pairs of known DTIs were collected, comprising 5089 drugs and 3286 targets. These curated interactions were treated as positive samples. The negative set was generated through random disruption of the positive set as is conventional in literature (Wen *et al.* 2017, Huang *et al.* 2021, Zhang *et al.* 2024, Zhao *et al.* 2025). Specifically, for each positive DTI pair, we selected the target and reassigned it to a different drug that is known to interact with another target, thereby forming a synthetic non-interacting drug–target pair. Negative samples were produced in a 1:1 ratio with the positive set, yielding 26 196 total pairs. In the drug cold-start case, there were 23 284 training samples, 1286 validation samples and 1626 testing samples. In the protein cold-start case, there were 23 684 training samples, 1164 validation samples and 1348 testing samples.

#### 2.2.3 Kiba

The KIBA (Tang *et al.* 2014) dataset tabulates experimental values for drug–target binding affinities, represented by the KIBA score. The threshold of 12.1 was used to curate a binary classification dataset from continuous affinity values (Öztürk *et al.* 2018, Zhao *et al.* 2025). 12 765 positive and 55 306 negative pairs were collected, comprising 133 drugs and 2106 targets.

### 2.3 Benchmarks

#### 2.3.1 MolTrans

MolTrans (Huang *et al.* 2021) operates on SMILES strings of drugs and amino acid sequences of proteins directly with a transformer model to encode feature embeddings. A Hadamard-product-based interaction matrix is constructed, and predictions are subsequently made by CNNs and FCNs.

#### 2.3.2 DLM-Dti

DLM-DTI (Lee *et al.* 2024) is a dual language model for the prediction of DTIs with hint-based learning. It comprises of the drug encoder, the target encoder, and the interaction prediction head. In the teacher model for target encoding, knowledge is distilled from the ProtBERT transformer. In the drug encoder, molecular representation vectors are obtained from SMILES sequences by employing the ChemBERTa encoder. Joint representation is achieved through concatenation and the prediction is made by a Fully Connected Layer.

#### 2.3.3 MocFormer

MocFormer (Zhang *et al.* 2024) is a proposed two-stage pretraining-driven transformer. Uni-Mol and ESM-2 are used to encode drug and protein features respectively. The representations are first pooled, then passed through multiple multi-head self-attention stacks for further feature extraction. Bilinear pooling is performed to model cross interactions, obtaining an interaction map along with a joint representation forwarded through a readout Fully Connected Layer.

### 2.4 Implementation and hyperparameters

3DICE was implemented in Python 3.12.10, except for the protein-side embeddings, which were precomputed in Python 3.8.20. A batch size of 32, learning rate of 5e−5, dropout of 0.3 and weight decay of 0 was used for 3DICE. Training was performed on a single NVIDIA RTX 5090 GPU with 32GB of VRAM for 60 epochs. Some embeddings were computed on an Apple M1 Pro. More details on the hyperparameters are provided in Appendix C, available as supplementary data at *Bioinformatics* online, while dependencies and package versions are listed on the GitHub repository. Details on the metrics used are provided in Appendix D, available as supplementary data at *Bioinformatics* online.

## 3 Results and discussion

### 3.1 3DICE achieves superior performance on benchmarks

The performance of 3DICE compared to other state-of-the-art models on the DrugBank, KIBA, and cold-start datasets is summarized in Table 1.

**Table 1:** Performance comparison among benchmarks on different datasets.

	ACC	PPV	TPR	F1	MCC	AUROC	AUPRC
DrugBank							
MolTrans	0.7780 (0.0063)	0.7437 (0.0128)	0.8723 (0.0202)	0.8027 (0.0025)	0.5587 (0.0045)	0.8650 (0.0031)	0.8520 (0.0073)
DLM-DTI	0.7906 (0.0016)	0.7854 (0.0078)	0.8000 (0.0154)	0.7920 (0.0042)	0.5818 (0.0029)	0.8651 (0.0035)	0.8605 (0.0030)
MocFormer	0.7972 (0.0021)	0.7741 (0.0030)	0.8463 (0.0045)	0.8081 (0.0027)	0.5976 (0.0029)	0.8816 (0.0019)	0.8780 (0.0071)
3DICE	0.8198 (0.0040)	0.8086 (0.0118)	0.8385 (0.0100)	0.8231 (0.0018)	0.6403 (0.0074)	0.8933 (0.0021)	0.8852 (0.0037)
KIBA							
MolTrans	0.8813 (0.0022)	0.7189 (0.0160)	0.6505 (0.0231)	0.6826 (0.0083)	0.6113 (0.0081)	0.9047 (0.0041)	0.7513 (0.0148)
DLM-DTI	0.8679 (0.0035)	0.6970 (0.0412)	0.5754 (0.0726)	0.6262 (0.0284)	0.5535 (0.0188)	0.8868 (0.0048)	0.7053 (0.0118)
MocFormer	0.8904 (0.0009)	0.7352 (0.0062)	0.6792 (0.0152)	0.7058 (0.0060)	0.6391 (0.0049)	0.9222 (0.0004)	0.7913 (0.0005)
3DICE	0.8915 (0.0006)	0.7645 (0.0175)	0.6373 (0.0263)	0.6984 (0.0099)	0.6357 (0.0058)	0.9231 (0.0009)	0.7941 (0.0016)
Drug cold-start							
MolTrans	0.6857 (0.0127)	0.6648 (0.0161)	0.6651 (0.0217)	0.6646 (0.0136)	0.3995 (0.0075)	0.7540 (0.0135)	0.7502 (0.0138)
DLM-DTI	0.7032 (0.0045)	0.7195 (0.0120)	0.6731 (0.0216)	0.6935 (0.0091)	0.4025 (0.0068)	0.7661 (0.0075)	0.7664 (0.0021)
MocFormer	0.6990 (0.0011)	0.6855 (0.0017)	0.7356 (0.0052)	0.7096 (0.0019)	0.3991 (0.0023)	0.7788 (0.0005)	0.7754 (0.0007)
3DICE	0.7474 (0.0058)	0.7471 (0.0207)	0.7434 (0.0351)	0.7445 (0.0101)	0.4913 (0.0130)	0.8195 (0.0044)	0.8182 (0.0052)
Protein cold-start							
MolTrans	0.6987 (0.0010)	0.6775 (0.0110)	0.6816 (0.0141)	0.6795 (0.0022)	0.3964 (0.0058)	0.7689 (0.0051)	0.7767 (0.0036)
DLM-DTI	0.7139 (0.0056)	0.7398 (0.0045)	0.6564 (0.0238)	0.6954 (0.0121)	0.4288 (0.0109)	0.7752 (0.0097)	0.7714 (0.0069)
MocFormer	0.7407 (0.0011)	0.7625 (0.0018)	0.6991 (0.0007)	0.7294 (0.0009)	0.4830 (0.0023)	0.8137 (0.0010)	0.8211 (0.0009)
3DICE	0.7476 (0.0103)	0.7737 (0.0218)	0.7053 (0.0211)	0.7375 (0.0053)	0.5048 (0.0095)	0.8243 (0.0032)	0.8297 (0.0035)

Five replications were performed (*n* = 5). Data is expressed as mean (std).

3DICE achieves excellent performance on the DrugBank dataset, outperforming the baselines across all reported metrics except for TPR. It is competitive even on KIBA, which is particularly challenging due to class imbalance. In the cold-start scenarios, expected performance degradations are observed for all models. Despite this, 3DICE outperforms all baselines across every reported metric on both the drug and protein cold-start settings, suggesting that its 3D structural encoders learn transferable stereochemical representations that generalize beyond seen compounds. This behaviour is particularly desirable in real-world drug discovery scenarios where predictions are often required for novel compounds lacking prior interaction data. The above results suggest that incorporating more informative feature representations and detailed interaction modelling contributes to improved performance.

To assess whether observed performance differences between models are statistically significant, we followed the method comparison protocol recommended by Ash *et al.* (2025): for each metric and dataset, a repeated measures ANOVA (RM-ANOVA) was first conducted across all four models using the five independent replications as subjects. Where the omnibus test indicated a statistically significant difference (α = 0.05), pairwise post-hoc comparisons between 3DICE and each baseline were performed using paired t-tests, with Holm–Bonferroni correction applied across the three comparisons to control the family-wise error rate. For our experiments, t-tests were performed for all replications as all were significant. Unstandardized effect sizes are reported as mean differences, where positive values indicate superior performance of 3DICE. Results are visualized as forest plots showing uncorrected 95% confidence intervals of the mean difference for descriptive purposes, and significance annotations reflect Holm-corrected *P-*value (*P < .05, **P < .001, ***P < .001). These are paired with Multiple Comparisons Similarity (MCSim) heatmaps, which quantify pairwise prediction agreement between models. Statistical test results for all datasets and metrics are provided in Appendix A (Fig. S1–S5, available as supplementary data at *Bioinformatics* online).

### 3.2 3DICE is computationally efficient

Beyond predictive performance, 3DICE also offers substantial efficiency gains over the baselines, as summarized in Table 2. This efficiency is a direct consequence of the architectural decision to delegate the learning of representations to massively pretrained 3D structural encoders, with the trainable prediction head being a very lightweight stack. With only 12.9 million trainable parameters, 3DICE is an order of magnitude smaller than MolTrans and MocFormer, and roughly a quarter the size of DLM-DTI. This compactness translates directly to lower memory consumption and higher inference throughput on identical hardware. We posit that the higher-fidelity representations produced by 3DICE’s encoders reduce the representational burden on the downstream prediction head, enabling competitive performance with substantially fewer parameters. In this sense, the efficiency advantage of 3DICE is also evidence for the value of 3D protein and drug structural representations in DTI prediction.

**Table 2:** Computational complexity comparisons.

Model	Trainable parameter count (M)	Peak training memory (MB)	Forward pass time (ms)	Training time (epoch) (s)
MolTrans	235	26 366	2.747	450
DLM-DTI	49.4	5212	1.961	174
MocFormer	349	15 292	0.185	13.1
3DICE	12.9	1758	0.051	7.73

All runs performed on RTX 5090 with batch size of 32.

### 3.3 Attention is not all you need for explanation

Many DTI studies interpret highly attended regions as evidence of binding relevance or mechanistic importance (Huang *et al.* 2021, Kurata and Tsukiyama 2022, Zeng *et al.* 2024). However, attention weights do not necessarily correspond to causal importance and may highlight spurious or inhibitory signals unrelated to the model’s decision process (Jain and Wallace 2019, Serrano and Smith 2019). We therefore evaluate the *faithfulness* of attention (Liu *et al.* 2022), defined as the extent to which highlighted regions genuinely influence model predictions.

Following the deletion-based framework of Liu *et al.* (2022), we progressively mask highly attended drug atoms or protein residues and measure the resulting change in model output. Two masking strategies are compared: *selective deletion*, where positions are removed in descending order of attention weight, and *random deletion*, which serves as a control.

To quantify prediction changes, we define the logit margin between the positive and negative classes as $m=z_{+}-z_{-}$. The change in margin after masking is:


(1) $$\Delta m=m^{\prime }-m_{0}=(z_{+}^{\prime }-z_{-}^{\prime })-(z_{+0}-z_{-0})$$

where $m_{0}$ and $m^{\prime }$ denote the margins before and after masking, respectively. Positive values of Δm indicate a shift toward the positive class, whereas negative values indicate a shift toward the negative class.

**Figure 2:**
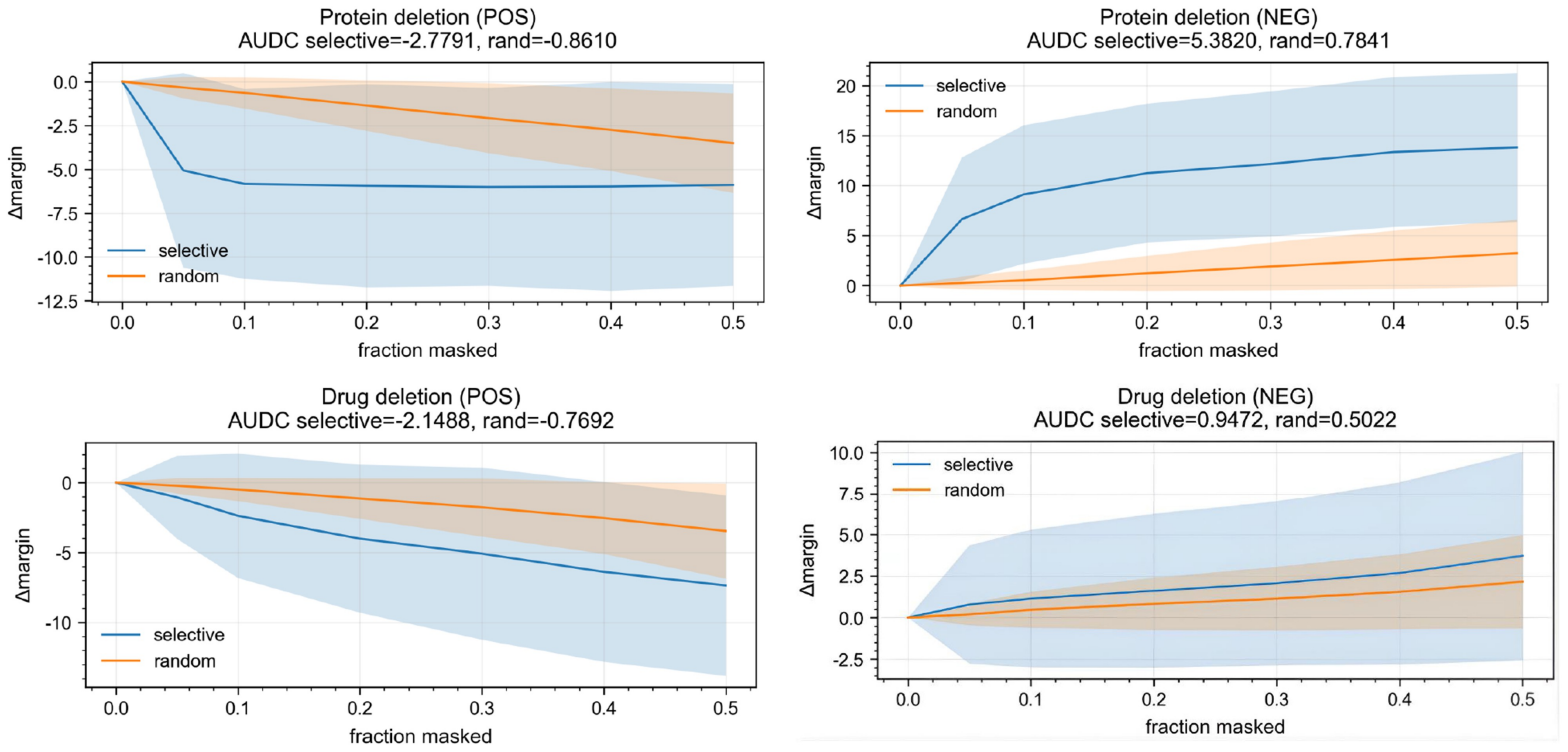
Deletion curves from faithfulness test.

We evaluated 512 positive and 512 negative samples. Figure 2 plots Δm as a function of the masked fraction, which we term the *Deletion Curve*. The Area Under the Deletion Curve (AUDC) is reported for both selective and random deletion.

For positive samples, selective deletion consistently produces a larger decrease in Δm than random deletion for both drugs and proteins, resulting in more negative AUDC values. This indicates that highly attended regions contribute substantially to positive predictions. The effect is particularly pronounced for proteins, where the selective curve drops sharply before gradually converging toward the random baseline, suggesting that a relatively small subset of residues carries most of the predictive signal.

For negative samples, drug deletion produces only minor changes in Δm, with similar AUDC values for selective and random masking. In contrast, selective masking of protein residues increases Δm more rapidly than random masking, indicating that these residues also contribute to negative predictions. Importantly, deletion curves alone do not establish whether such residues encode positive or negative binding evidence. Rather, they demonstrate that the highlighted regions are influential to the model’s decision. We therefore further investigate the biological significance of these sites in the following section.

### 3.4 Key site investigation

**Figure 3:**
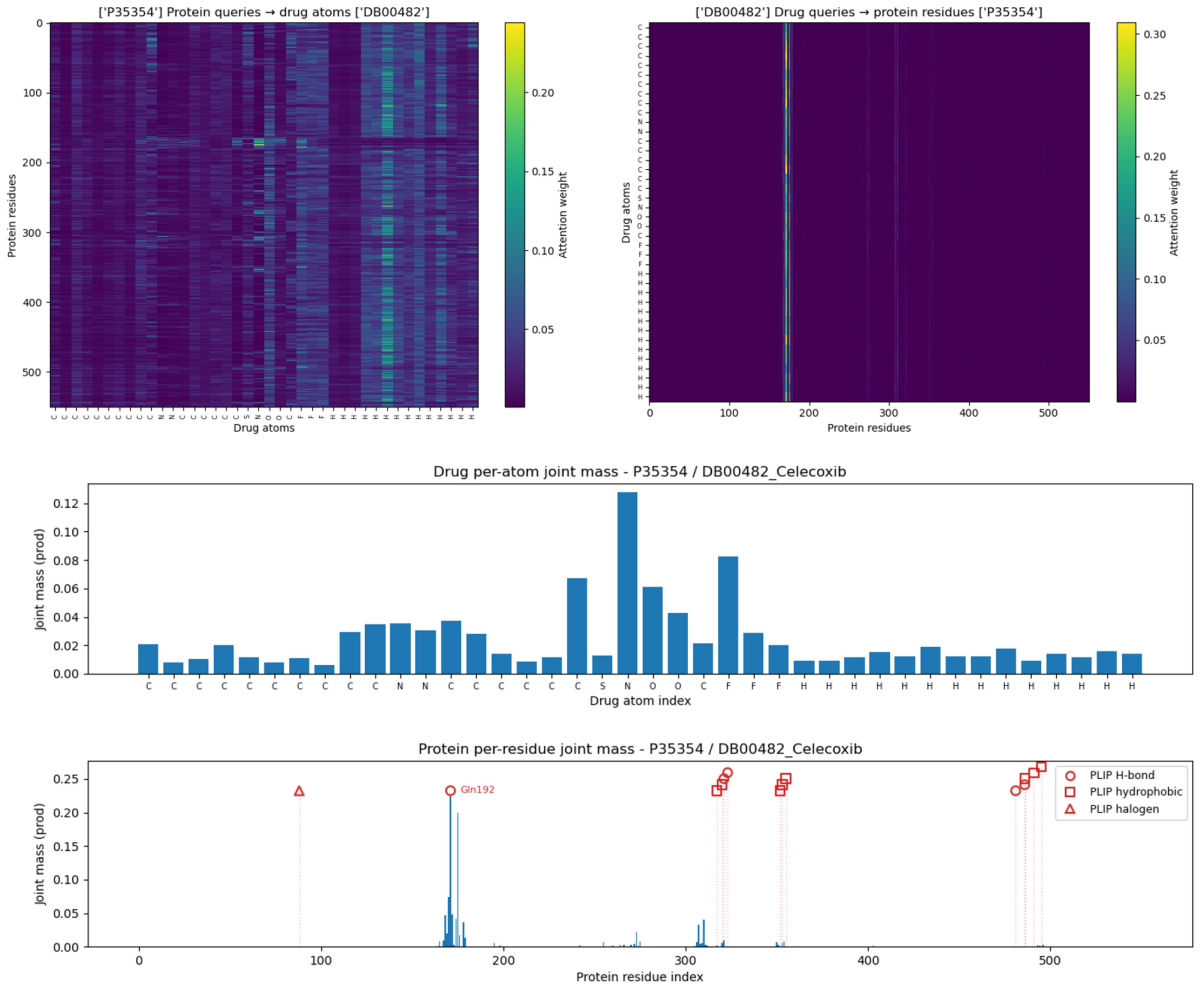
Attention weights and joint scores in positive samples.

To further examine the biological relevance of the learned attention patterns, we analysed a well-characterized DTI pair: celecoxib (DrugBank ID: DB00482), a selective non-steroidal anti-inflammatory drug, and prostaglandin-endoperoxide synthase 2 (PTGS2/COX-2; UniProt ID: P35354). The pair was passed through 3DICE and the two co-attention matrices were extracted: the protein-to-drug matrix, in which residues attend over atoms, and the drug-to-protein matrix, in which atoms attend over residues. The resulting attention maps are shown in Fig. 3.

Protein attention was concentrated within a small number of residues, whereas drug attention was more broadly distributed across the molecular structure. Furthermore, residues receiving high attention typically corresponded to more localized atom-level attention patterns, suggesting stronger residue–atom specificity. For example, atom N20 exhibited strong coupling to a restricted subset of residues, whereas atom H32 appeared broadly important but less specific.

To facilitate interpretation, we computed a joint co-attention mass by taking the elementwise product of the two attention matrices and summing across rows or columns to obtain global importance scores for individual atoms and residues. This score emphasizes mutually reinforced residue–atom interactions and provides a more interpretable representation of cross-modal coupling.

**Figure 4:**
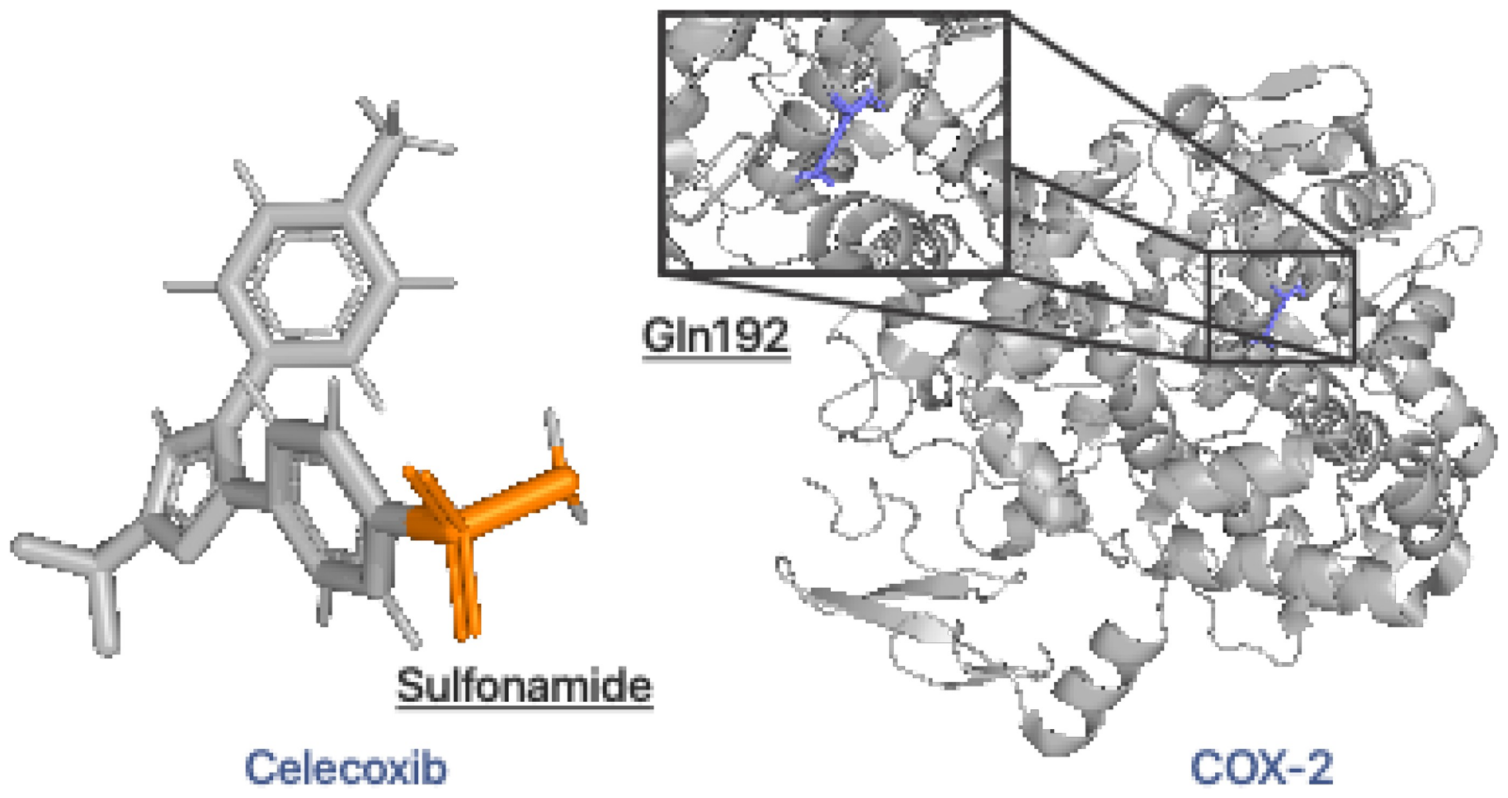
Identified binding sites.

Because experimentally resolved protein structures may not perfectly align with canonical FASTA sequences, structural residue indices were mapped back to FASTA coordinates prior to analysis. As an experimental reference, we additionally performed PLIP (Schake *et al.* 2025) analysis on the murine COX-2–celecoxib co-crystal structure (PDB: 3LN1), which identified 13 contact residues. These residues are overlaid in Fig. 4.

The largest residue-attention peak occurred near structural index 175, corresponding to the vicinity of Gln192. On the drug side, atom N20 corresponds to the sulfonamide nitrogen of celecoxib. Previous crystallographic and redocking studies have consistently reported interactions between the sulfonamide group and the polar pocket region surrounding Gln192 (Rowlinson *et al.* 2003, Al-Ghulikah *et al.* 2022). Additional peaks were observed near Leu352, Tyr385, and Trp387, all of which are established components of the celecoxib binding pocket. These findings suggest that the co-attention mechanism is capable of recovering biologically meaningful interaction motifs. However, not all known contact residues were strongly highlighted; for example, Arg513 (Al-Ghulikah *et al.* 2022) did not appear among the dominant peaks.

**Figure 5:**
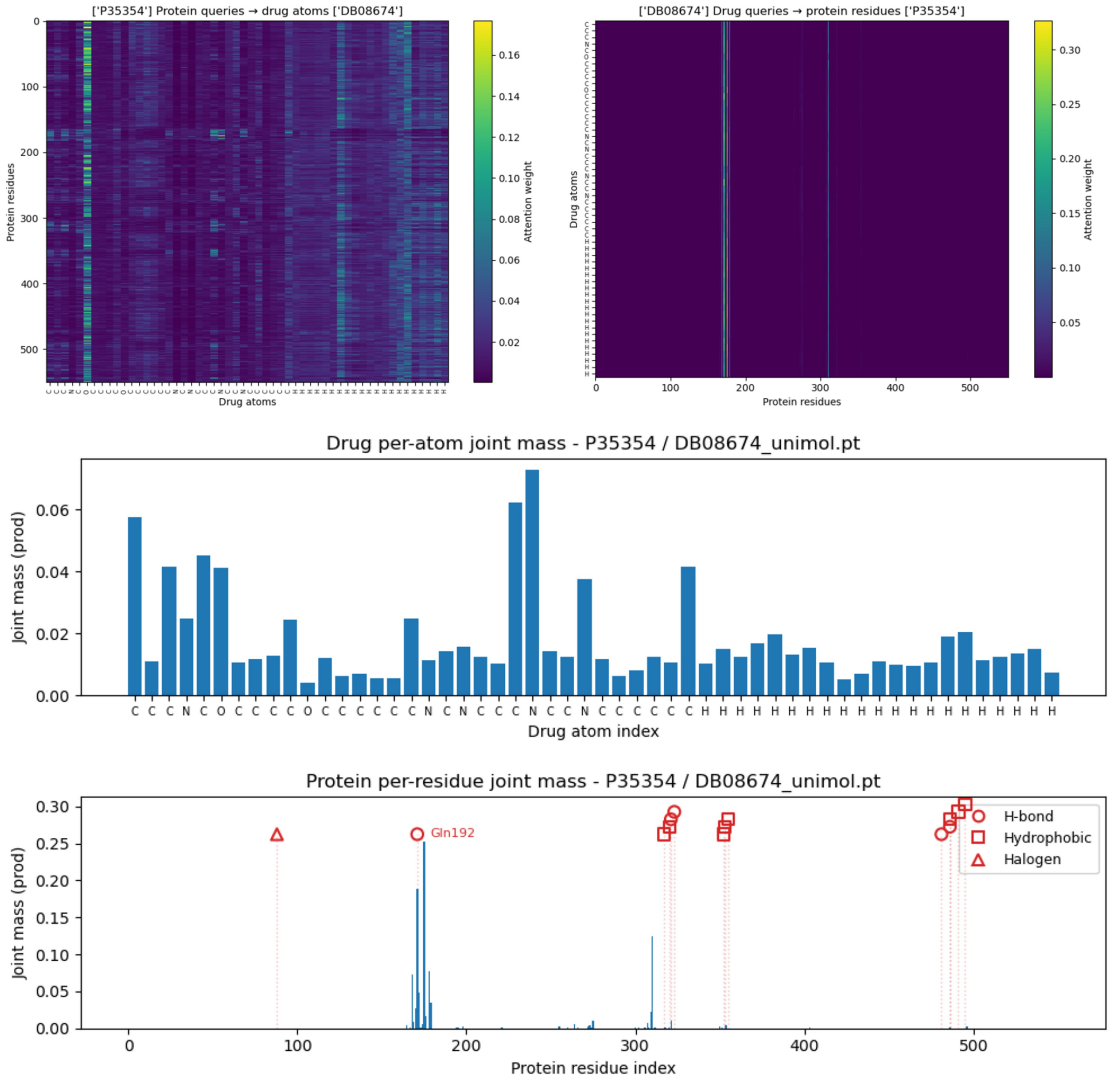
Attention weights and joint scores in negative samples.

We also observed weaker peaks near FASTA indices 257 and 292, which are not canonical celecoxib-contact residues. Inspection of the corresponding attention maps revealed no strong association with individual drug atoms, suggesting that these regions may encode broader structural or contextual information rather than direct chemical contacts. To further investigate this behaviour, we repeated the analysis on a negative sample (Fig. 5).

Interestingly, similar protein regions remained highlighted in both positive and negative samples. Combined with the faithfulness analysis, this observation suggests that attention primarily identifies residues that are informative to the model’s decision process rather than residues that intrinsically encode positive or negative binding effects. The downstream classifier subsequently determines how information from these regions contributes to the final prediction.

Importantly, the faithfulness experiments demonstrated that masking these residues significantly altered model confidence, indicating that they carry genuine predictive signal. Nevertheless, predictive importance should not be equated with direct physical interaction. The highlighted residues may correspond to true binding contacts, structural determinants, or broader protein signatures that assist classification.

Overall, our findings support a nuanced interpretation of attention. In some cases, attention successfully recovers experimentally supported interaction motifs, such as the Gln192–sulfonamide interaction. However, attention also highlights regions that contribute to prediction for reasons beyond direct physical contact. This observation is consistent with previous reports that attention-based explanations only partially overlap with experimentally verified binding residues (Kurata and Tsukiyama 2022). Therefore, while attention maps provide useful mechanistic hypotheses, claims regarding binding-site identification should be supported by additional structural or experimental validation.

### 3.5 Ablation study

In our ablation study, we evaluate the effectiveness of the CNN and co-attention (CA) components (Table 3). For the CNN variants, the CNN encoder was replaced with a self-attention (SA) module comparable to those used in state-of-the-art transformer-based DTI models. Because ESM-2 produces 2048-dimensional embeddings, unlike the 512-dimensional outputs of ESM-IF1, mean pooling was used to project the features to a lower dimension so the downstream prediction head can be retained.

**Table 3:** Ablation study on the DrugBank dataset.

	ACC	PPV	TPR	F1	MCC	AUROC	AUPRC
3DICE w/o CA	0.8091 (0.0017)	0.7889 (0.0067)	0.8555 (0.0079)	0.8155 (0.0015)	0.6199 (0.0025)	0.8787 (0.0049)	0.8675 (0.0051)
3DICE Prot SA	0.8104 (0.0061)	0.7940 (0.0213)	0.8407 (0.0421)	0.8157 (0.0106)	0.6237 (0.0120)	0.8850 (0.0055)	0.8769 (0.0037)
3DICE Drug SA	0.8157 (0.0042)	0.7937 (0.0076)	0.8533 (0.0158)	0.8224 (0.0052)	0.6334 (0.0090)	0.8913 (0.0035)	0.8851 (0.0024)
3DICE ESM-2	0.8101 (0.0040)	0.7790 (0.0107)	0.8663 (0.0120)	0.8202 (0.0023)	0.6243 (0.0065)	0.8907 (0.0036)	0.8855 (0.0063)
3DICE (base)	0.8198 (0.0040)	0.8086 (0.0118)	0.8385 (0.0100)	0.8231 (0.0018)	0.6403 (0.0074)	0.8933 (0.0021)	0.8852 (0.0037)

Five replications were performed (*n* = 5). Data is expressed as mean (std).

Despite ESM-2 being a newer and larger model, ESM-IF1 performs better in this context, showing that the inclusion of 3D representation provides significant tangible benefits.

### 3.6 Conformer stability analysis

A limitation of 3DICE is that it takes one conformer as input for the drug structural representation. Since RDKit uses a stochastic conformer generation method, there is a risk that a single sampled conformer may not adequately represent the bioactive conformation, particularly for flexible molecules. To assess the sensitivity of 3DICE to conformer seed choice, we conducted a conformer stability analysis motivated by McNutt *et al.* (2023). For each drug in the test set, *K* = 20 alternative conformers were generated using the same ETKDG + MMFF94 pipeline as in training, varying only the random seed, and inference was performed with frozen model weights. The number was chosen in accordance with McNutt’s recommendation that fewer than 25 conformers is sufficient for most structure-based tasks.

As shown in Table S6, using an alternative conformer seed produces no statistically significant change in hard-prediction metrics (ACC: *t* = −0.884, *P* = .427; MCC: *t* = −1.19, *P* = .301), but a highly significant decrease in AUROC (*t* = −23.9, *P* = .0000181), with an overall prediction flip rate of 5.45%. The difference in degradations across metrics is because hard metrics like ACC and MCC are only determined by which side of the decision threshold each prediction falls on, and so are only affected when conformer perturbation actually flips a prediction. AUROC, by contrast, depends on the rank ordering of predicted probabilities across the entire test set, making it sensitive to small shifts in confidence even when the label is preserved.

The performance decrease under seed perturbation likely reflects a mild distributional shift in Uni-Mol embeddings between conformer seeds, as the 3DICE prediction head is implicitly optimized on the embedding characteristics of the training seed. However, consistent with McNutt *et al*.’s finding that small ensembles improve over single conformers, majority voting across K seeds largely recovers and even exceeds the training seed on hard-prediction metrics, demonstrating that the mild conformer sensitivity of 3DICE is readily mitigated through a simple ensemble strategy without retraining.

### 3.7 Generalization and robustness

A key concern in DTI prediction and PCM modelling is that models may achieve strong benchmark performance by treating protein identity as a categorical label rather than genuinely learning transferable structural features. This phenomenon, often referred to as the Clever Hans effect, has been documented across a range of deep learning models for protein-ligand affinity prediction (Roth and Bajorath 2025, Bajorath 2026).

To assess whether 3DICE exhibits the same behaviour, we conduct the following experiment inspired by Atas Guvenilir and Doğan (2023). On the DrugBank and derived cold-start datasets, we train separate model variants in which either the Uni-Mol drug embeddings or the ESM-IF1 protein embeddings are replaced globally with random vectors throughout training and evaluation. These random vectors are sampled from a Gaussian distribution matched to the empirical mean and per-dimension standard deviation of the real training embeddings. The results are presented in Table 4.

**Table 4:** Clever Hans test on different datasets.

	ACC	PPV	TPR	F1	MCC	AUROC	AUPRC
DrugBank							
Drug Noise	0.6091 (0.0034)	0.6123 (0.0074)	0.5955 (0.0151)	0.6037 (0.0043)	0.2183 (0.0069)	0.6492 (0.0030)	0.6485 (0.0032)
Protein Noise	0.5774 (0.0059)	0.6118 (0.0285)	0.4368 (0.0729)	0.5060 (0.0370)	0.1633 (0.0182)	0.6013 (0.0021)	0.6269 (0.0009)
Baseline	0.8198 (0.0040)	0.8086 (0.0118)	0.8385 (0.0100)	0.8231 (0.0018)	0.6403 (0.0074)	0.8933 (0.0021)	0.8852 (0.0037)
Drug cold-start							
Drug Noise	0.5832 (0.0041)	0.5730 (0.0057)	0.6540 (0.0235)	0.6106 (0.0084)	0.1683 (0.0081)	0.6286 (0.0042)	0.6223 (0.0022)
Protein Noise	0.5080 (0.0050)	0.5493 (0.0440)	0.1021 (0.0301)	0.1704 (0.0404)	0.0291 (0.0217)	0.5285 (0.0031)	0.5248 (0.0091)
Baseline	0.7474 (0.0058)	0.7471 (0.0207)	0.7434 (0.0351)	0.7445 (0.0101)	0.4913 (0.0130)	0.8195 (0.0044)	0.8182 (0.0052)
Protein cold-start							
Drug Noise	0.5129 (0.0045)	0.5596 (0.0264)	0.1311 (0.0618)	0.2072 (0.0766)	0.0409 (0.0119)	0.5263 (0.0130)	0.5359 (0.0077)
Protein Noise	0.5611 (0.0063)	0.5697 (0.0151)	0.5059 (0.0399)	0.5349 (0.0166)	0.1234 (0.0144)	0.5799 (0.0026)	0.6057 (0.0023)
Baseline	0.7476 (0.0103)	0.7737 (0.0218)	0.7053 (0.0211)	0.7375 (0.0053)	0.5048 (0.0095)	0.8243 (0.0031)	0.8297 (0.0035)

Five replications were performed (*n* = 5). Data is expressed as mean (std).

In contrast with the findings of Atas Guvenilir and Doğan, where PCM models trained with random protein vectors retained much of the full model’s performance on the random split, 3DICE shows substantial degradation under all six scrambling conditions across our three stratified splits. Despite a degree of memorization still being present, this indicates that the model does not rely on a single modality as a memorization shortcut to the degree reported in prior literature. The collapse to near-chance performance on the cold-start datasets when the non-cold-start modality is scrambled further indicates that these splits introduce minimal signal leakage between train and test, validating our splitting strategy.

### 3.8 Limitations

The availability of 3D structures and associated computational demands remain long-standing challenges that hinder the proliferation of 3D structural encoders. Although this work demonstrates its feasibility and benefits, it is important to acknowledge the challenges that were encountered during the investigation process. In particular, the reliance on experimentally resolved or reliably predicted 3D structures resulted in the loss of a substantial portion of samples from the DrugBank and KIBA datasets due to missing structures or parsing failures. These limitations in 3D structure availability are expected to diminish with the evolution of protein-folding models. Additionally, precomputing and storing embeddings and full 3D structures introduces additional computational and storage overheads. While this is somewhat offset by the lightweight prediction head of our model, the practical cumbersomeness of dealing with 3D structures must nevertheless be acknowledged.

A further limitation concerns conformer sampling, which was investigated previously. Averaging Uni-Mol embeddings across multiple conformers per molecule, or ensembling models trained using different conformer seeds, is a natural extension that could yield more robust and representative drug representations.

Finally, it is worth contextualizing 3DICE within the broader landscape of DTI prediction approaches, as the most appropriate method depends on the application’s requirements. Docking and physics-based methods, while computationally intensive at scale, offer direct mechanistic explainability that attention-based interpretability does not fully replicate. Recent advances in high-throughput docking have also partially mitigated their scalability disadvantage (Fraser *et al.* 2025). 3DICE and related ML approaches are best suited to large-scale virtual screening where speed and throughput are paramount; they are complementary to structure-based methods in settings where mechanistic detail is critical. The appropriate choice of method depends on the stage of the discovery pipeline, the available computational resources, and the degree of mechanistic insight required.

## 4 Conclusion

DTI prediction remains a critical component of modern drug discovery, where accurate computational methods can accelerate screening and reduce experimental costs. In this study, we proposed 3DICE, an interpretable DTI prediction framework that combines pretrained 3D structural encoders (Uni-Mol and ESM-IF1) with explicit co-attention-based interaction modelling. By leveraging rich geometric representations of both drugs and proteins, 3DICE captures biologically meaningful cross-modal interactions while maintaining computational efficiency.

Extensive benchmarking demonstrated that 3DICE consistently achieves strong performance across standard and cold-start DTI prediction tasks, outperforming or matching state-of-the-art approaches. Beyond predictive accuracy, we performed complementary faithfulness, conformer stability, statistical significance, and modality-scrambling analyses, providing a rigorous assessment of interpretability, robustness, and generalization. Our findings indicate that attention highlights decision-relevant regions but should be interpreted as an indicator of model focus rather than a direct map of physical binding sites.

Overall, 3DICE demonstrates that integrating pretrained 3D structural representations with explicit interaction modelling provides an effective and interpretable framework for DTI prediction. We anticipate that this approach will contribute to the development of more robust AI-driven tools for accelerating drug discovery and virtual screening.

## Supplementary Material

btag488_Supplementary_Data

## Data Availability

Our model and dataset are freely available at: https://github.com/austinatose/3DICE.
