# Supplementary material for: 3DICE: interpretable 3D cross-modal learning for drug–target interaction prediction and large-scale drug discovery
Source: Bioinformatics. 2026 Jul 2;42(7):btag488. doi: 10.1093/bioinformatics/btag488 (PMC13384055; doi:10.1093/bioinformatics/btag488)
Supplement: btag488_Supplementary_Data [file btag488_supplementary_data.pdf]

Appendix

Appendix A: Statistical Tests and Results

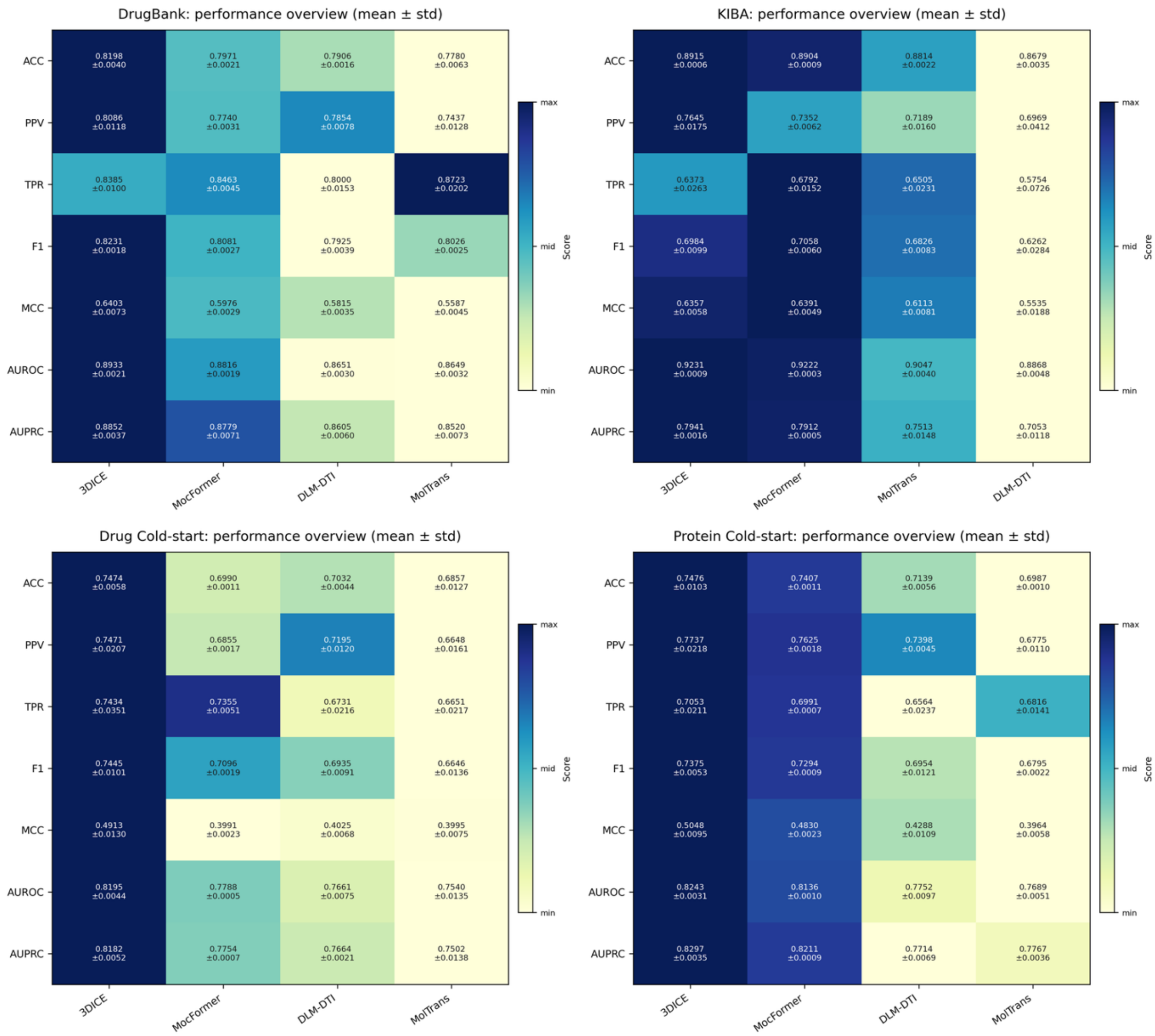

Figure S1: Results Summary

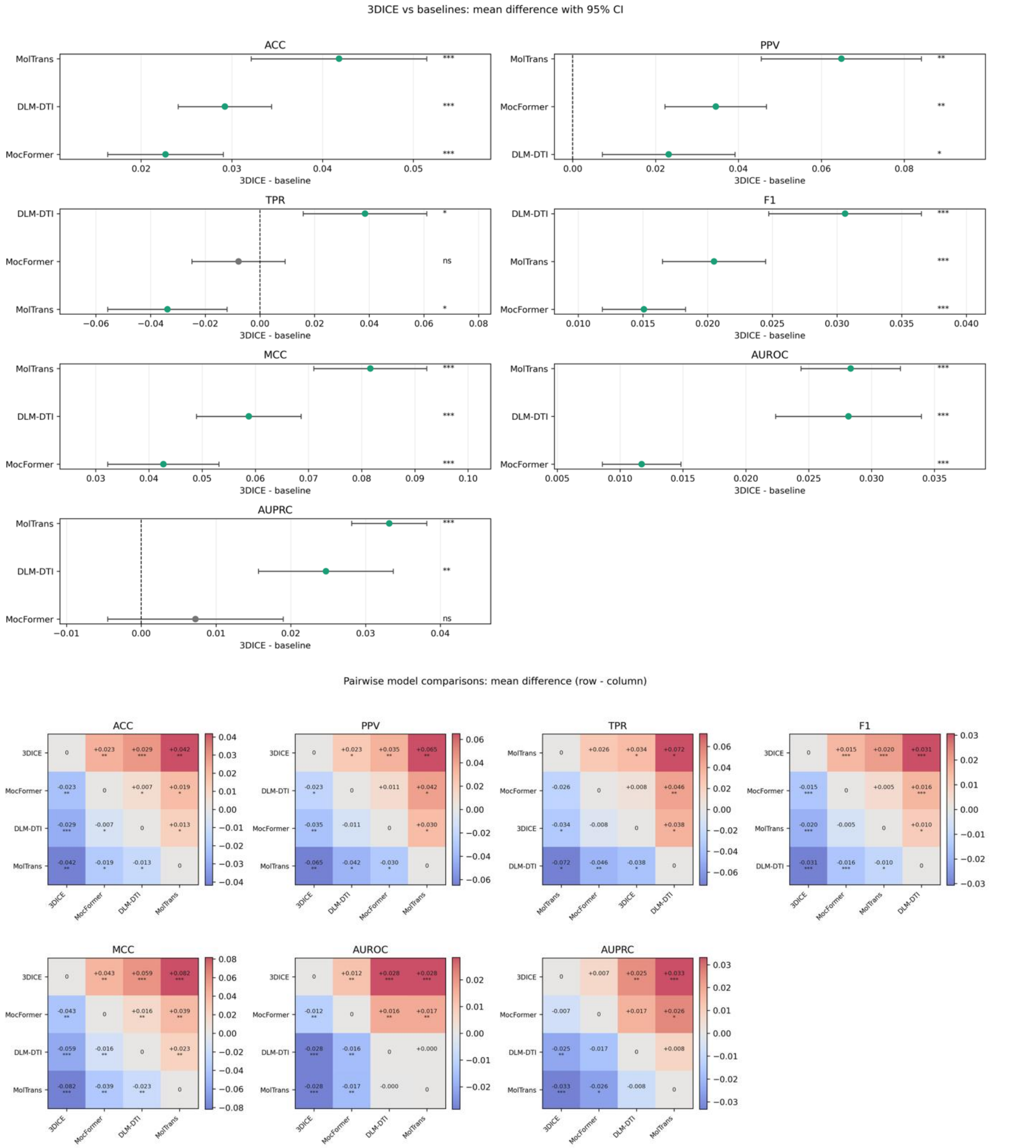

Figure S2: Statistical tests for DrugBank dataset

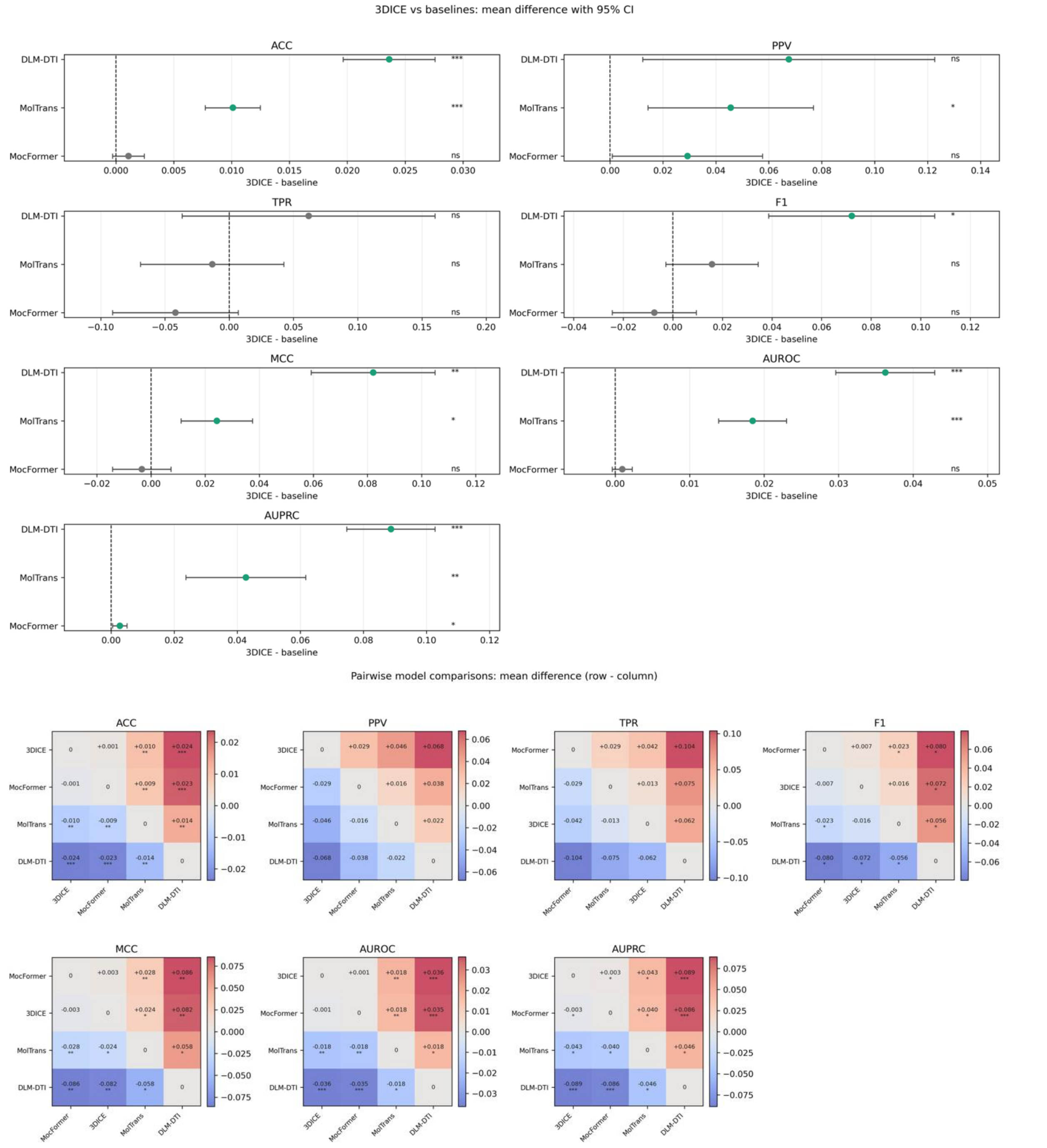

Figure S3: Statistical tests for KIBA statistics

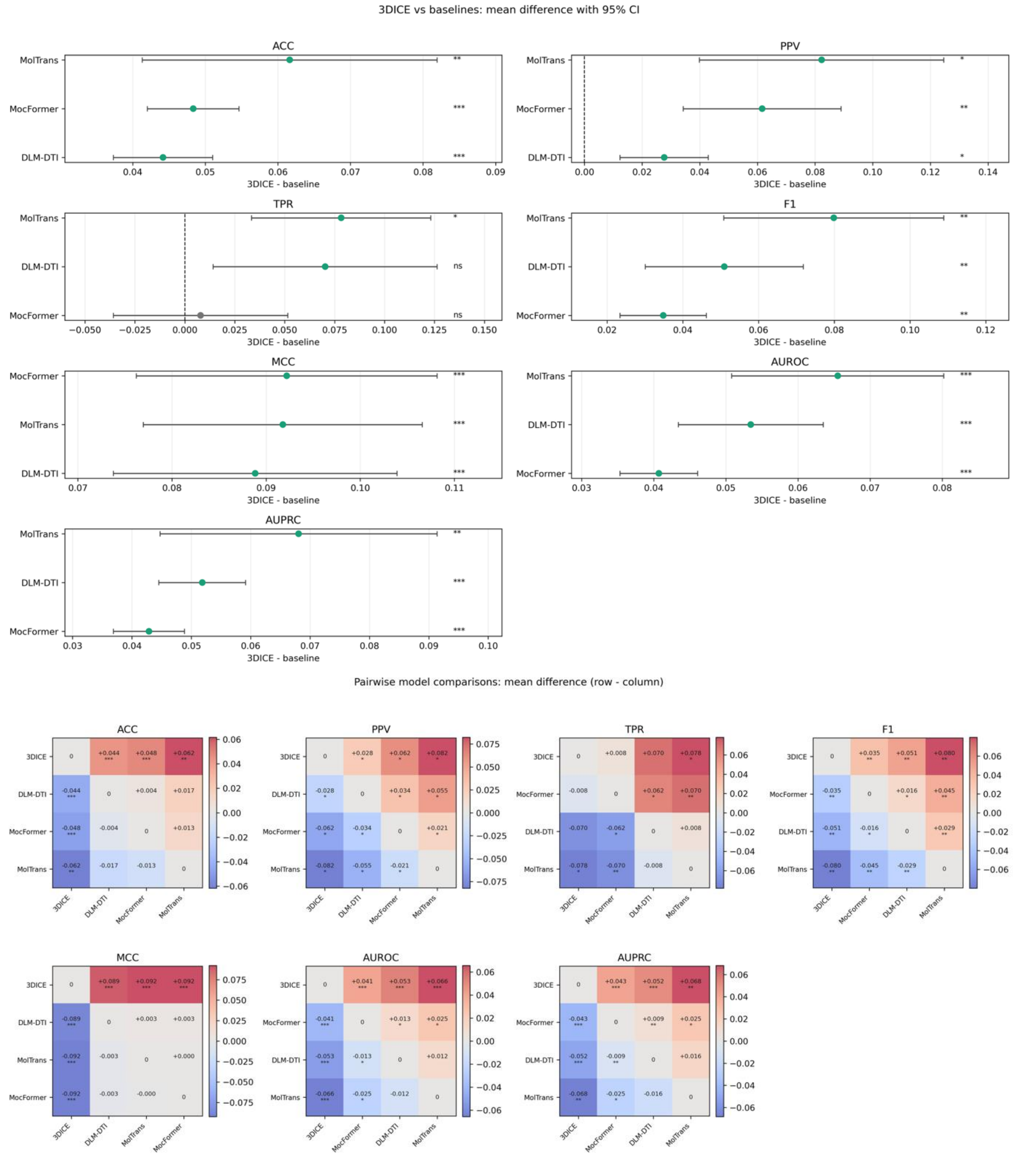

Figure S4. Statistical tests for Drug cold-start statistics

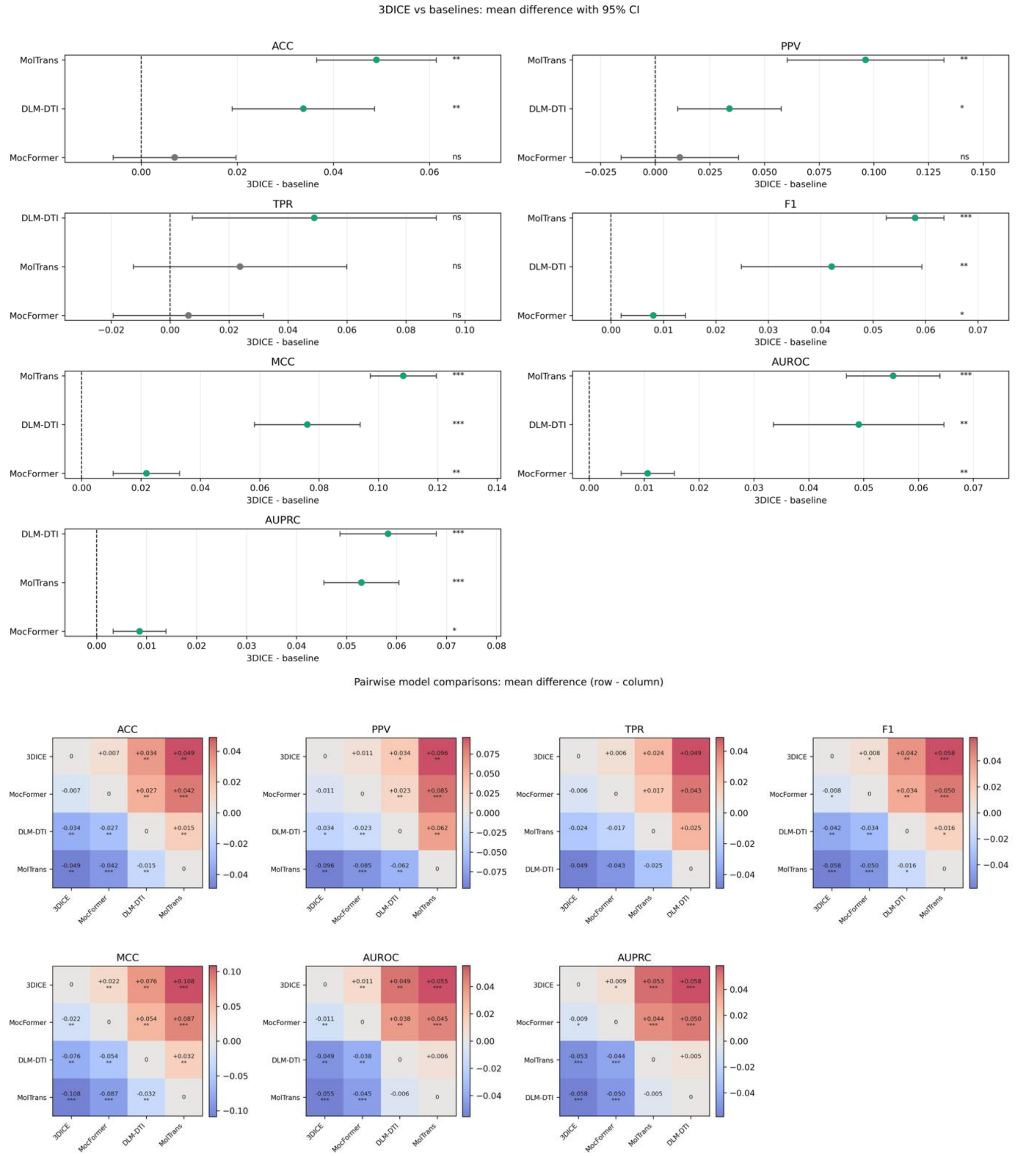

Figure S5. Statistical tests for Protein cold-start statistics

Table S6. Conformer Stability Analysis on the DrugBank dataset

	ACC	MCC	AUROC
Training Seed	0.81984<br>(0.00167)	0.64030<br>(0.00644)	0.89329<br>(0.00415)
Random Seed	0.81894<br>(0.01279)	0.62679<br>(0.02546)	0.86399<br>(0.00274)
Delta	-0.00090<br>(-0.02093, 0.01082)	-0.01351<br>(-0.04512, -0.01810)	-0.02930<br>(-0.03270, -0.02590)
Majority Vote	0.83268<br>(0.02227)	0.66292<br>(0.04605)	0.86694<br>(0.02210)

Training seed and majority vote values are mean (std) across n = 5 model replications. For random seed values, for each of K = 20 conformer seeds, metrics were averaged across n = 5 model replications; mean (std) then reported across K = 20 seed-averaged values. Delta reported as random seed value – training seed value (95% CI).

Appendix B: Architectural Details

Protein Feature Encoder

The frozen pretrained model Evolutionary Scale Modelling – Inverse Folding 1 (ESM-IF1) was used which produced a $L_p \times 512$ feature representation.

$$\boldsymbol{X} \in \mathbb{R}^{L_p \times 512}$$

We apply a 2-layer 1D convolutional neural network (CNN) across the sequence dimension to capture local substructural patterns.

$$\text{Block}(\boldsymbol{A}) = \boldsymbol{A} + \text{Dropout}\Big(\text{ReLU}\big(\text{Conv1D}(\boldsymbol{A})\big)\Big)$$

$$\text{CNN}(\boldsymbol{A}) = \text{Block}_2\big(\text{Block}_1(\boldsymbol{A})\big)$$

$$\boldsymbol{X}^{(1)} = \text{CNN}(\boldsymbol{X})$$

Drug Feature Encoder

The frozen pretrained model Uni-Mol was used which produced a $L_d \times 512$ feature representation.

$$\boldsymbol{Y} \in \mathbb{R}^{L_d \times 512}$$

A similar CNN was applied:

$$\boldsymbol{Y}^{(1)} = \text{CNN}(\boldsymbol{Y})$$

Co-attention Module

A co-attention module was implemented to explicitly model interactions between proteins and drugs via bidirectional cross-attention. Each cross-attention block once again follows a pre-norm Transformer-style architecture with residual connections, dropout, and a position-wise feed-forward network.

Multi-head attention was implemented for this component. For each head $i \in \{1, \ldots, H\}$ we first form:

$$\text{Head}_i(\boldsymbol{Q}_i, \boldsymbol{K}_i, \boldsymbol{V}_i) = \text{Softmax}\left(\frac{(\boldsymbol{Q}_i \boldsymbol{W}_i^Q)(\boldsymbol{K}\boldsymbol{W}_i^K)^\top}{\sqrt{\frac{d}{H}}}\right)(\boldsymbol{V}\boldsymbol{W}_i^V)$$

We then define the MultiHeadAttention function:

$$\text{MultiHeadAttention}(\boldsymbol{Q}, \boldsymbol{K}, \boldsymbol{V}) = \text{concat}\big(\text{Head}_i(\boldsymbol{Q}_i, \boldsymbol{K}_i, \boldsymbol{V}_i), \dots, \text{Head}_{\text{H}}(\boldsymbol{Q}_H, \boldsymbol{K}_H, \boldsymbol{V}_H)\big) \cdot \boldsymbol{W}_0$$

Next, the feed-forward network:

$$\text{FFN}(\boldsymbol{A}) = \text{ReLU}(\boldsymbol{A}\boldsymbol{W}_1 + \boldsymbol{b}_1)\boldsymbol{W}_2 + \boldsymbol{b}_2$$

On the protein side:

$$\boldsymbol{O}_p = \boldsymbol{X}^{(1)} + \text{Dropout}\left(\text{MultiHeadAttention}\left(\text{LN}_p^{(1)}\left(\boldsymbol{X}^{(1)}\right), \boldsymbol{Y}^{(1)}, \boldsymbol{Y}^{(1)}\right)\right)$$

$$\boldsymbol{O}_p^{(1)} = \boldsymbol{O}_p + \text{Dropout}\left(\text{FFN}_p\left(\text{LN}_p^{(2)}(\boldsymbol{O}_p)\right)\right)$$

And similarly for drugs:

$$\boldsymbol{O}_d = \boldsymbol{Y}^{(1)} + \text{Dropout}\left(\text{MultiHeadAttention}\left(\text{LN}_d^{(1)}\left(\boldsymbol{Y}^{(1)}\right), \boldsymbol{X}^{(1)}, \boldsymbol{X}^{(1)}\right)\right)$$

$$\boldsymbol{O}_d^{(1)} = \boldsymbol{O}_d + \text{Dropout}\left(\text{FFN}_d\left(\text{LN}_d^{(2)}(\boldsymbol{O}_d)\right)\right)$$

$\boldsymbol{O}_p^{(1)}$ and $\boldsymbol{O}_d^{(1)}$ represent attended protein and drug features. Padding masks are applied during attention to account for different lengths.

Fusion Module

Mean pooling was applied to the attended protein and drug representation matrices to get a fixed-length representation vector. Mean pooling was applied, then were each passed through a fully connected layer that reduced their dimensions from 512 to 256:

$$\boldsymbol{P} = \text{MeanPool}\left(\boldsymbol{O}_p^{(1)}\boldsymbol{W}_p + \boldsymbol{b}_p\right), \boldsymbol{D} = \text{MeanPool}\left(\boldsymbol{O}_d^{(1)}\boldsymbol{W}_d + \boldsymbol{b}_d\right)$$

where $\boldsymbol{W}_p, \boldsymbol{W}_d \in \mathbb{R}^{256\times512}$. Finally, a concatenation forms the final joint representation vector.

$$\boldsymbol{Z} = [\boldsymbol{P} \parallel \boldsymbol{D}] \in \mathbb{R}^{512}$$

Readout MLP

The fused representation is forwarded to a fully connected multilayer perceptron (MLP) that outputs two logits corresponding to the positive and negative interaction. The larger of the two logits was taken to be the predicted class.

$$\boldsymbol{R} = \text{MLP}(\boldsymbol{Z}) \in \mathbb{R}^2$$

Appendix C: Hyperparameters

Parameter	Value
Epoch	60
Batch	32
Optimiser	Adam
Learning rate	5e-5
Weight decay	0
Dropout	0.3
Activation Function	ReLU
CNN Kernel Size	3
CNN Layer Count	2
CNN Dimensions	[512, 512]
Attention head number	4
Feed-forward Dimension	2048
Pooling method	Mean Pooling
Reduction MLP Dimension	256
Readout MLP Dimensions	[1024, 1024, 512, 2]

Appendix D: Performance Metrics

Let TP, TN, FP, and FN denote the number of true positives, true negatives, false positives, and false negatives respectively. The following metrics were used and labelled as follows:

Accuracy (ACC) measures the overall proportion of correctly classified samples:

$$\mathrm{ACC} = \frac{\mathrm{TP} + \mathrm{TN}}{\mathrm{TP} + \mathrm{TN} + \mathrm{FP} + \mathrm{FN}}$$

Precision (PPV) measures the proportion of predicted positive samples that are truly positive:

$$\mathrm{PPV} = \frac{\mathrm{TP}}{\mathrm{TP} + \mathrm{FP}}$$

Recall (TPR) measures the proportion of true positive samples that are correctly identified:

$$\mathrm{TPR} = \frac{\mathrm{TP}}{\mathrm{TP} + \mathrm{FN}}$$

F1-score is the harmonic mean of precision and recall:

$$\mathrm{F1} = 2 \cdot \frac{\mathrm{PPV} \cdot \mathrm{TPR}}{\mathrm{PPV} + \mathrm{TPR}}$$

Matthews Correlation Coefficient (MCC) provides a balanced measure of classification quality that accounts for all four confusion matrix terms:

$$\mathrm{MCC} = \frac{\mathrm{TP} \cdot \mathrm{TN} - \mathrm{FP} \cdot \mathrm{FN}}{\sqrt{(\mathrm{TP} + \mathrm{FP})(\mathrm{TP} + \mathrm{FN})(\mathrm{TN} + \mathrm{FP})(\mathrm{TN} + \mathrm{FN})}}$$

Area Under Receiver Operating Curve (AUROC) measures the model's ability to discriminate between positive and negative samples across varying decision thresholds.

Area Under Precision-Recall Curve (AUPRC) measures the trade-off between precision and recall across thresholds.
